# An Expanded Set of Amino Acid Analogs for the Ribosomal Translation of Unnatural Peptides

**DOI:** 10.1371/journal.pone.0000972

**Published:** 2007-10-03

**Authors:** Matthew C. T. Hartman, Kristopher Josephson, Chi-Wang Lin, Jack W. Szostak

**Affiliations:** Howard Hughes Medical Institute, Department of Molecular Biology, Center for Computational and Integrative Biology, Simches Research Center, Massachusetts General Hospital, Boston, Massachusetts, United States of America; Baylor College of Medicine, United States of America

## Abstract

**Background:**

The application of *in vitro* translation to the synthesis of unnatural peptides may allow the production of extremely large libraries of highly modified peptides, which are a potential source of lead compounds in the search for new pharmaceutical agents. The specificity of the translation apparatus, however, limits the diversity of unnatural amino acids that can be incorporated into peptides by ribosomal translation. We have previously shown that over 90 unnatural amino acids can be enzymatically loaded onto tRNA.

**Methodology/Principal Findings:**

We have now used a competition assay to assess the efficiency of tRNA-aminoacylation of these analogs. We have also used a series of peptide translation assays to measure the efficiency with which these analogs are incorporated into peptides. The translation apparatus tolerates most side chain derivatives, a few α,α disubstituted, *N*-methyl and α-hydroxy derivatives, but no β-amino acids. We show that over 50 unnatural amino acids can be incorporated into peptides by ribosomal translation. Using a set of analogs that are efficiently charged and translated we were able to prepare individual peptides containing up to 13 different unnatural amino acids.

**Conclusions/Significance:**

Our results demonstrate that a diverse array of unnatural building blocks can be translationally incorporated into peptides. These building blocks provide new opportunities for in vitro selections with highly modified drug-like peptides.

## Introduction

The recent development of translation systems reconstituted entirely from purified components [1]–[4] has enabled the ribosomal synthesis of peptides composed primarily of unnatural (i.e. non-proteinogenic) amino acids. The major roadblock to the ribosomal synthesis of highly modified drug-like peptides is the limited number of unnatural building blocks known to be compatible with the translation apparatus. This dearth of unnatural building blocks results in part from difficulties in loading unnatural amino acids onto tRNA, the key first step in translation. Several techniques for charging tRNAs with unnatural amino acids have been developed such as chemoenzymatic tRNA acylation [5]–[7], ribozyme acylation [8], chemical acylation using PNA thioesters [9], and the use of engineered aminoacyl-tRNA synthetases (AARSs) [10]. Unfortunately all of these approaches require specialized reagents and/or multistep syntheses. In contrast, an all-enzymatic system using the wild-type AARSs would be as convenient as a standard translation experiment using the twenty proteinogenic amino acids. To this end we have recently developed a MALDI-TOF MS screening assay [11] that has uncovered over 90 unnatural building blocks that are AARS substrates. The incorporation of these amino acids into peptides, however, depends on the efficiency of aminoacylation, and on whether or not they are compatible with the translational steps subsequent to aminoacylation. The specificity of many of these steps with respect to unnatural amino acids is unknown. It is clear; however, that the ribosome does not tolerate certain very sterically demanding aminoacyl-tRNAs [12], [13], and recent studies suggest that a threshold EF-Tu affinity is required for aminoacyl-tRNAs to enter the A-site of the ribosome [14]. Particular types of backbone analogs also seem to be excluded during these translational steps [15], although exactly how they are rejected is not well-understood.

Our newly defined pool of AARS substrates is ideal for addressing these specificity questions because it contains a diverse collection of side chain, *N*-methyl, α,α-disubstituted, and β-aminoacyl-tRNAs. Here we describe a straightforward means of determining whether an enzymatically charged unnatural amino acid can be effectively incorporated into a peptide. The results of these experiments have allowed us to identify over 50 unnatural amino acids that can be incorporated at a single defined position in a peptide with high efficiency using an all-enzymatic translation system. Our work identifies building blocks that can be combined to prepare highly modified peptides that contain multiple unnatural amino acids. We also identify many cases where the engineering of synthetases, EF-Tu or the ribosome itself may be required to enable efficient incorporation of interesting analogs.

## Results

### Aminoacylation competition assay

**Figure 1:**
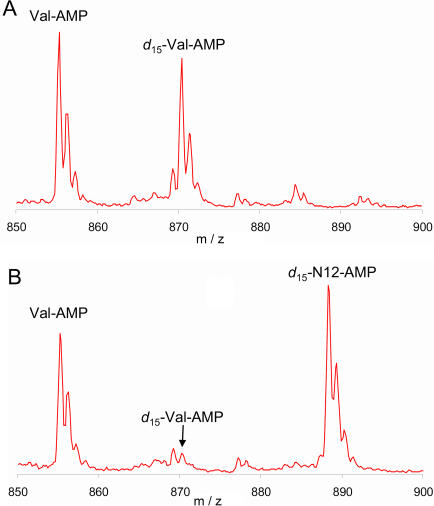
Inhibition of the charging of valine by analog N15. The intensity of the *d*_15_-Val-AMP in the absence (A) or presence (B) of analog N15 (50 mM) is shown. Valine was present in both assays at 50 µM. The difference in the ratio of Val-AMP and *d*_15_-Val-AMP in the assays was used for the inhibition calculation. As expected for a ValRS substrate, a peak corresponding to the *d*_15_-N15-AMP was also observed in the analog-containing assay. This peak was not used in the determination of inhibition.

The MALDI-TOF MS assay that we used to identify unnatural amino acids that are AARS substrates [11] does not provide a quantitative measure of tRNA charging efficiency. The rate of synthesis of aminoacylated tRNA, and the degree to which an analog competes effectively against traces of contamination with the natural amino acid, can greatly affect the yield and purity of analog-containing peptides synthesized by in vitro translation. However, detailed kinetic analysis of large numbers of analogs is not feasible. We therefore devised an AARS competitive inhibition assay in which the charging of a natural amino acid in the presence and absence of an analog could be quantitatively compared by MS through the use of an isotopically labeled derivatizing reagent [11]. In order to compare analog inhibition among different AARSs, we set the natural amino acid at roughly 2x its *K_m_* and then added the unnatural amino acid analog at 1000-fold higher concentration. As an example, the assays for the inhibition measurement of valine analog 3-fluoro-valine (N15) are shown in Figure 1. For each assay an identical amount of non-isotopically derivatized Val-AMP was added as an internal standard, and experiments in the presence or absence of the analog were performed using the deuterium (*d*_15_) labeled derivatizing reagent [11]. The presence of competing N15 clearly led to a strong decrease in the intensity of the *d_15_*-Val-AMP peak (Figure 1B) when compared to the assay with valine alone (Figure 1A). Comparison of the ratio of Val-AMP to *d_15_*-Val-AMP in the presence and absence of the analog allowed determination of the % inhibition value.

The inhibition data for the analogs is presented in Table 1. As expected, different analogs vary widely in their ability to competitively inhibit the aminoacylation of the natural amino acids. In general, we expect good competitive inhibitors to be good substrates, although this correlation need not always hold. Nevertheless, as discussed below, analogs that are good competitive inhibitors tend to be translated more efficiently than analogs that are poor competitors, supporting the idea that the synthesis of high levels of aminoacylated tRNA during a translation reaction facilitates the synthesis of analog-bearing peptides.

**Table 1:** AARS competition data for unnatural amino acids

Analog^a^	% Inhibition^b^	Range	Analog^a^	% Inhibition^b^	Range	Analog^a^	% Inhibition^b^	Range
A1	20	0.4	C8	76	1.1	P1	3	13.2
A2	49	3.8	C9	15	8.4	P2	4	7.8
A3	46	23.0	C10	82	10.3	P3	−5	7.8
A4	−14	13.0	C11	−3	14.2	P4	−1	1.8
A5	78	2.1	C12	−4	2.1	P5	92	2.9
A6	11	2.2	C13	−1	7.7	P6	1	1.0
A7	24	1.8	N1	82	3.3	P7	70	0.0
A8	−5	2.1	N2	63	2.4	α1 (LeuRS)	14	1.8
A9	94	6.1	N3	73	0.5	α1 (ValRS)	59	3.3
A13	85	1.9	N4	34	5.5	α2 (LeuRS)	2	9.7
A14	24	11.2	N5	70	3.0	α2 (ValRS)	58	2.4
A15	52	1.2	N6	97	0.5	α3	31	5.4
A16	13	3.8	N9	−8	2.4	α4	9	2.0
A17	111	9.8	N10	2	2.4	α5	1	1.2
A18	76	11.4	N11	9	0.8	α6	32	1.3
A19	24	22.9	N12	83	1.4	α7	0	5.4
A20	77	11.3	N13	80	0.4	β1	3	1.0
A21	−9	16.1	N14	76	3.0	β3	−17	1.0
A22	−7	3.8	N15	74	1.4	β7	12	6.3
A23	19	2.7	N16 (LeuRS)	53	7.8	β8	5	6.8
A24	7	4.1	N16 (ValRS)	37	1.2	β9	−5	5.1
A25	3	6.5	N17	83	16.7	β10	−7	11.8
C1	9	0.1	N18	52	22.2	M1	14	3.1
C2	12	2.0	N19	−8	19.7	M2	0	11.0
C3	78	3.6	N21	39	0.3	M3	27	1.7
C4	77	0.6	N22	30	12.7	M4	12	6.9
C5	85	0.0	N23	67	12.6	M5	−19	4.4
C6	59	0.5	N24	28	10.0	M6	−2	5.8
C7	135	2.0						

a Analog abbreviations are taken from ref. 11. Structures of all analogs are listed in the subsequent tables.

b % Inhibition is the average loss in intensity of the AA-AMP peak relative to a control with no analog added; higher values signify more inhibition.

### Translation efficiency of analogs

**Figure 2:**
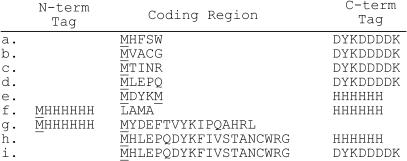
Peptides encoded by mRNA templates used in this study. The position(s) of the radiolabeled methionine or histidine are underlined.

We have previously defined a set of ∼90 amino acid analogs that can be enzymatically charged onto tRNA [11]. To determine which of these could be incorporated into peptides, we used an assay based on the translation of short peptides containing a single unnatural amino acid. We prepared 5 separate mRNA templates containing a C-terminal FLAG or His_6_ tag for purification; this set of templates is similar to but more complete than that previously described [1]. Each template was designed to test analogs of 4 of the natural amino acids (Figure 2, *a–e*). The translation reactions were then carried out in the PURE translation system [1], [4] with ^35^S-methionine as the N-terminal radiolabel, except for Met analogs which were tested using ^3^H-His and template e. In each test, one of the natural amino acids was omitted and a corresponding analog added in its place; thus synthesis of radiolabeled tagged peptide required readthrough of the codon assigned to the unnatural amino acid [1]. The peptide yield for each unnatural amino acid was determined relative to the peptide yield for the corresponding natural amino acid, by comparison of the peptide radioactivity for the all-natural peptide with that containing the unnatural analog. To verify that the translated peptide contained the unnatural amino acid, the mass of the peptide was determined by MALDI-MS. For unnatural amino acids that differed in mass by +/−1 amu from their natural counterparts, we verified incorporation using templates requiring multiple (4–7) incorporations. For instance, incorporation of the tryptophan analog A18 was tested using a template with 4 tryptophan codons (see supporting information Figure S1) and was unambiguously identified due to its +4 mass difference from the tryptophan containing peptide.

We grouped the amino acid analogs that we studied into three classes based on translation efficiency and specificity. Class I monomers resulted in a peptide yield that was >75% of that obtained with the corresponding natural amino acid, and the translated peptide was homogeneous by MALDI-MS analysis. Class II monomers were translated less efficiently, with a peptide yield ranging from 25% to 75% of that obtained with the natural amino acid, but the translated peptide was still homogeneous. Class III monomers were either poorly translated ( <25% peptide yield) and/or led to a heterogeneous mixture of peptides. In many cases inefficient translation correlated with poor aminoacylation, suggesting that the synthesis of aminoacylated tRNA may have limited peptide yield. Peptide heterogeneity was most often due to the misincorporation of natural amino acids in place of the desired analog. We discuss these analogs in groups based on their side chain and backbone compositions. We used the same amino acid abbreviations used in our previous paper [11] (C = Charged, P = Polar, A = Aromatic, N = Nonpolar, α =  α,α disubstituted, β = beta amino acid, M = *N*-methyl). Since our first report we have found two additional unnatural amino acids that are efficiently aminoacylated and translated; these compounds (‘Photo-Leu’, N25; ‘Photo-Met’, N26) are also discussed below. Finally, we have described the translation results for twelve of these analogs in a preliminary report [1]; because these analogs are included in the other experiments described herein, we have included them here.

### Charged side chain analogs

**Figure 3:**
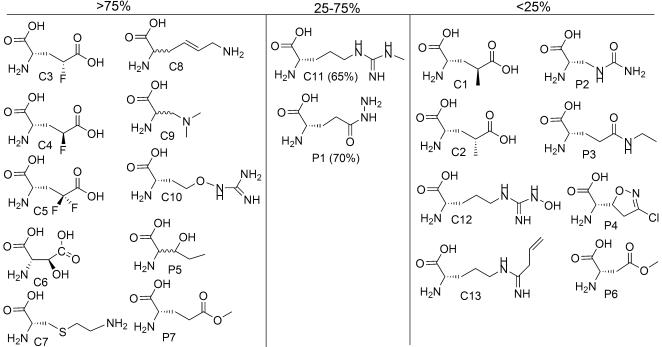
Amino acids with charged or polar side chains and their translation yields relative to the cognate natural AA.

Most of the negatively charged GluRS and AspRS substrates shown in Figure 3 were efficient translation substrates. All three 4-fluoro-glutamates (C3–C5) were efficiently incorporated, but both 4-methyl analogs (C1 and C2) were poorly translated. Amino acids C1 and C2 also compete much more poorly with glutamic acid for the GluRS active site than their fluoro counterparts, suggesting that inefficient charging is the cause of their poor translation.

l- *threo*-*β*-hydroxy aspartic acid (C6) is an Asp analog found in certain NRPs, such as the iron-chelating aquachelins [16] and pyoverdins [17]. As observed previously, C6 was efficiently incorporated into peptides in place of Asp [1], [18]; it also competes well with Asp in the tRNA-Asp aminoacylation assay.

We have confirmed the efficient translation of the Lys analog, *S*-2-aminoethyl cysteine (C7) [1], [19], and observed that another Lys analog, trans-dehydro lysine (C8), was translated with high efficiency. C8 is incorporated into collagen in a chick embryo system [20]; both C7 and C8 exhibit antibacterial activity due to their ability to bind and inhibit bacterial riboswitches involved in lysine biosynthesis [21].

The positively charged Leu analog aza-leucine (C9) was a strong competitive inhibitor of LeuRS, and was also translated efficiently. Incorporation of this analog into protein is consistent with several reports, including upregulation of heat-shock genes when *E. coli* is challenged with aza-leucine [22], [23].

Of the four arginine analogs found to be AARS substrates, l-canavanine (C10) [1], [24] was translated efficiently, while l-NMMA (N^G^-methyl arginine) (C11) was translated with somewhat lower efficiency (65% peptide yield relative to Arg). N^G^-hydroxy arginine (C12) and vinyl-L-NIO (C13) did not yield any detectable peptide, and were also poor competitors in the AARS assay.

### Polar, uncharged

Two polar but uncharged amino acid analogs are excellent translation substrates (Figure 3). Of these, we and others had previously observed dl- *β*-hydroxy norvaline (P5) to be an excellent threonine surrogate in translation reactions [1], [24]. Remarkably, the l-Glu γ-methyl ester (P7) efficiently substituted for methionine in all steps of translation; fully formylated peptides containing the expected double incorporation of P7 were observed in experiments with template *e*. This analog is found in the microviridins, peptides produced by certain cyanobacteria, which were initially isolated as elastase inhibitors [25]. In contrast, the l-Asp *β*-methyl ester (P6) led to the translation of a heterogeneous mixture of peptides, including the non-formylated peptide and peptides that initiated at codons other than methionine, suggesting that the initiation complex does not tolerate this analog.

Of the three analogs previously found to be GlnRS substrates (P1–P3), l-glutamic acid γ-hydrazide [18], [26], l-albizziine [18], and l-theanine[18], only the hydrazide was tolerated by the translation apparatus. Acivicin (P4), the cyclic anti-cancer agent and γ-glutamanyl transpeptidase inhibitor [27] was not translated efficiently, consistent with its poor activity in the IleRS competition assay.

### Aromatic Analogs

**Figure 4:**
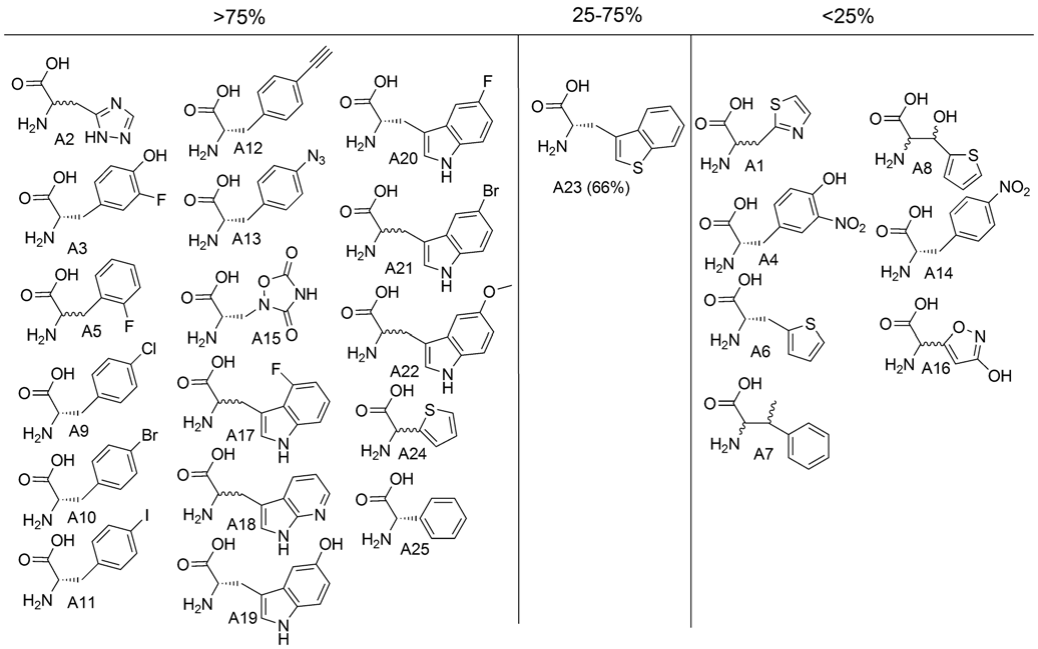
Amino acids with aromatic side chains and their translation yields relative to the cognate natural AA.

We examined two histidine analogs, *β*-2-thiazolyl-alanine (A1) and *β*-(1,2,4-triazol-3-yl-alanine) (A2) [28]–[30]; A2 allowed efficient peptide synthesis as described [1], but we could not detect any peptide product containing A1 (Figure 4). A1 also showed poorer competitive inhibition of HisRS (Table 1). Our observations are entirely consistent with earlier in vivo experiments in which a His-auxotrophic *E. coli* strain was used to show that A2, but not A1, could be incorporated into a specific protein [28].

The tyrosine analog 3-fluoro tyrosine [24], [31] (A3) was translated efficiently, as previously observed [1]. However, 3-nitro tyrosine (A4), with a bulkier substituent at the 3-position, was not detectably incorporated into peptides. Again, these results correlate with those of the AARS competition assay.

Of the four phenylalanine surrogates previously shown to be PheRS substrates [11], 2-fluoro Phe (A5), 2-thienyl Ala (A6) [24], *β*-methyl Phe (A7), and *β*-thienyl Ser (A8), only the previously described A5 [1] was translated efficiently. Use of the PheRS A294G mutant allowed several previously described *p*-substituted analogs (A9–A13) [32] chloro, bromo, iodo, ethynyl, and azido, to be introduced into peptides in good yields. However, *p*-nitrophenylalanine (A14) was poorly translated in the presence of this synthetase. Each of the poorly translated Phe analogs were also poor competitors in the AARS assay (all showed less than 25% inhibition).

All of the tryptophan analogs examined (A17–A23) proved to be competent translation substrates, despite a wide variation in AARS competition ability. There is some disagreement in the literature [33], [34] whether 3-(thianaphthen-3-yl)-l-alanine (A23) can be translated; in our system it was a reasonably good substrate.

The aromatic glutamate receptor agonists [35], [36] and GluRS substrates l-quisqualic acid (A15) and ibotenic acid (A16) had different fates in translation—only l-quisqualic acid was translated in good yields. Ibotenic acid was also much weaker in the AARS competition assay.

Both of the aromatic isoleucine surrogates dl-α-(2-thienyl)glycine (A24) and l-phenylglycine (A25) were excellent translation substrates, consistent with the successful in vitro suppression experiments with l-phenylglycine [37].

### Nonpolar

**Figure 5:**
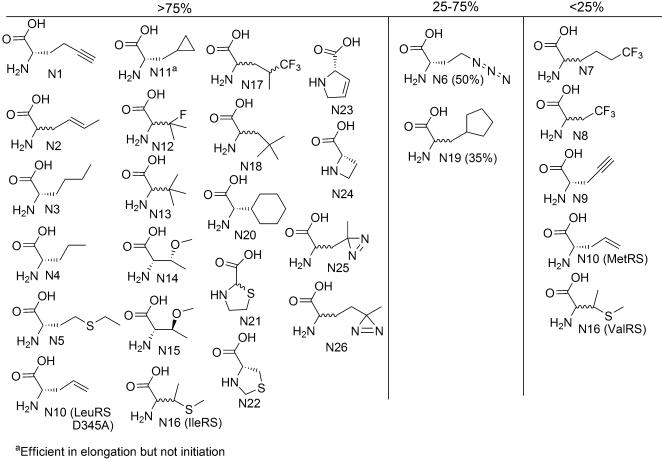
Amino acids with nonpolar side chains and their translation yields relative to the cognate natural AA.

A wide variety of nonpolar substrates were translated efficiently (Figure 5). We examined a large number of Met analogs using template e, ^3^H-His for labeling, and the His_6_ tag for peptide purification. We confirmed the translatability of many previously reported methionine analogs [1], [38]–[40] in our assay including 2-amino hex-5-ynoic acid, crotylglycine, l-norleucine, l-norvaline, l-ethionine, and l-β-azidohomoalanine, and (N1-N6). On the other hand, four other MetRS substrates, 6,6,6-trifluoronorleucine (N7), 2-amino-4,4,4-trifluorobutyric acid (N8), l-C-propargyl glycine (N9) [41], and l-allyl glycine (N10) [41] resulted in poor translation initiation and/or elongation without any evidence for misincorporation. Analog N10 (as well as N6) [1], [42] could, however, be charged and incorporated using the editing deficient LeuRS D345A [43]. β-Cyclopropyl alanine (N11) was efficiently translated, but the resulting peptide was a mixture of full length peptide and peptide lacking the N-terminal residue, suggesting initiation with N11 was difficult. The photoactive diazirine bearing Met analog (N26) [44] was efficiently incorporated into peptide. This analog, also known as photo-Met, should be a useful probe for studying the binding sites of in vitro translated peptides on protein targets.

Valine analogs 3-fluoro-valine (N12), *t*-butyl-glycine (N13), and *O-*methyl-l-threonine (N14) as well as (2S,3S)-2-amino-3-methoxybutanoic acid (N15) were incorporated into peptides in excellent yields. The analog 4-thia-isoleucine (N16) was charged by both ValRS and IleRS, but only the aminoacylated tRNA^Ile^ resulted in efficient analog incorporation into peptide. It remains unclear as to whether this difference reflects a difference in tRNA charging efficiency (the AARS competition assay showed they were both moderate competitors) or a difference in the interaction of the charged tRNAs with EF-Tu and/or the ribosome. The isoleucine analog l-cyclohexylglycine (N20) was easily integrated into peptides.

We and others have previously reported that translation reactions containing the leucine analog 5′,5′,5′-trifluoro leucine (N17) were high yielding [1], [24]. LeuRS substrates β-*t*-butyl-alanine (N18) and β-cyclopentyl alanine (N19) also could be translated in moderate to excellent yields. The photoreactive diazirine bearing Leu analog N25 is aminoacylated by LeuRS and efficiently incorporated into peptides. This analog (photo-Leu) [44] provides an additional probe of the interaction of translated peptides with protein targets.

All four proline analogs: thiazolidine-2-carboxylic acid (N21) [45] thiazolidine-4-carboxylic acid (N22) [46], 3,4-dehydro proline (N23) [47], [48], and l-azetidine-2-carboxylic acid (N24) [1], [49], were excellent substrates for the translation apparatus.

### α,α Disubstituted Amino Acids

**Figure 6:**
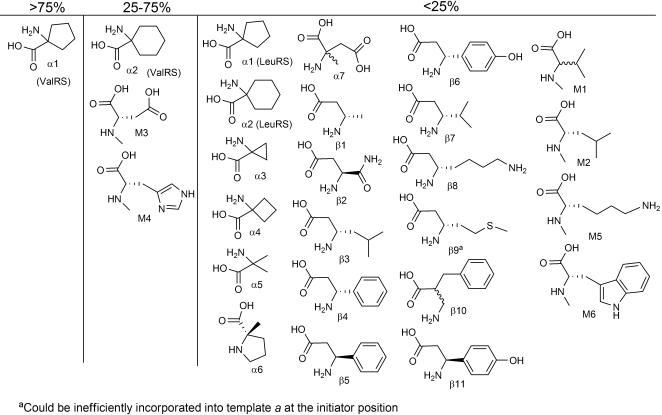
Backbone-modified amino acids and their translation yields relative to the cognate natural AA.

After completing our analysis of side chain analogs, we proceeded to test those backbone analogs that can be enzymatically charged onto tRNA, including α,α disubstituted amino acids, *β*-amino acids, *N*-methyl amino acids and α-hydroxy acids (Figure 6). Only two of the seven α,α disubstituted amino acids that are AARS substrates could be incorporated into peptides during translation (Figure 6, α1–α7). These two, 1-amino cyclopentanoic acid (α1) and 1-aminocyclohexanoic acid (α2), were both charged by both ValRS and LeuRS, and both were good translation substrates in response to Val but not Leu codons. Interestingly, α1 and α2 were also the best AARS competitors among the α,α disubstituted amino acids in the competition assay, suggesting that α,α disubstituted AAs incorporation into peptide in our system is controlled at least in part by the AARS. The ribosome and/or EF-Tu are also implicated in low translation efficiencies for α-methyl substituted AAs by a recent study using chemically acylated tRNA [15].

### β-amino acids

Although one β_2_ (β10) and eleven β_3_ (β1–β9, β11) amino acids are known to be AARS substrates [11], none were incorporated into peptides by the translation apparatus (Figure 6). Although this is a disappointing result, it is consistent with previous reports of poor to undetectable incorporation of β-amino acids in translation experiments [7], [15], [50], [51]. Literature reports do suggest that β-amino acids can act as peptide donors at the ribosome P site [52]. This is consistent with our finding that β9 is able to serve as an initiator amino acid, albeit inefficiently. Very recently, Sando and coworkers have found that β−hydroxy acids are competent translation substrates and have suggested that the elevated pKa of the amine in β−amino acids prevents deprotonation and peptidyl transfer [53]. If this interpretation is correct, ribosome engineering may be required to enable the efficient incorporation of β-amino acids into peptides.

One difficulty with our studies of the aminoacylation of the β-amino acids is that some of them have an identical mass to their natural analogs (β2, β4–β6, and β11). Since it is impossible to rule out natural amino acid contamination using our MALDI-MS assay for tRNA aminoacylation, it was possible that these analogs were not actually charged. However, the observation that they are not translated confirms that they are charged onto tRNA, since tRNA charging with contaminating natural amino acid would have led to efficient peptide translation.

### *N*-methyl amino acids

The *N*-methyl amino acids are of particular interest since they are very common in the natural product non-ribosomal peptides, to which they are thought to contribute protease resistance and membrane permeability. Of the six *N-*methyl amino acids that were AARS substrates (M1–M6, Figure 6) only *N-*methyl Asp (M3) and *N-*methyl His (M4) resulted in even moderate yields of translated peptide. One of the difficulties in testing the translation of these compounds was that many of them were contaminated with very small amounts of the unmethylated, natural amino acid. In order to remove this contamination an excess of the *N-*methyl amino acid was put into an AARS reaction. Since the contaminating natural amino acid is a more efficient substrate than the *N-*methyl analog and is at a much lower concentration, most of the natural amino acid became attached to the tRNA and was removed by EtOH precipitation [54]. The *N-*methyl AA containing EtOH layer was kept and evaporated. After repeating this procedure twice, formation of the natural amino acid-containing peptides was greatly suppressed. Unfortunately, the yields of *N*-methyl amino acid containing peptides remained low for M1, M2, M5 and M6, possibly due to the synthesis of limiting amounts of aminoacylated tRNA by the synthetase enzymes as evidenced by the poor ability of all of the *N*-methyl AAs to serve as competitors in our AARS assay.

### α-hydroxy carboxylic acids

**Figure 7:**
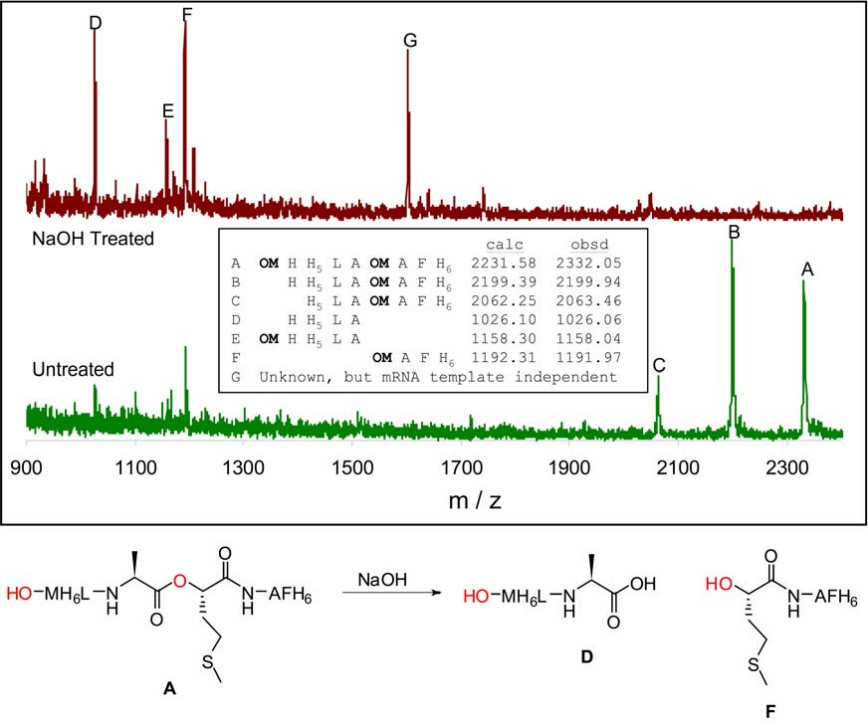
Ester hydrolysis shows that *α*-hydroxy methionine is incorporated. Top–Comparison of the peptide masses before and after treatment with NaOH which shows the expected hydrolyzed products labeled D, E and F. Bottom—Expected products from the hydrolysis of peptide containing an *α*-hydroxy methionine residue.

Alpha-hydroxy acids are another interesting class of backbone analogs that are also the defining component of the depsipeptides, a subset of the natural product non-ribosomal peptides. Every substitution of an amide linkage for an ester linkage in a depsipeptide removes one hydrogen bond donor from the molecule, which may contribute significantly to membrane permeability [55]. The incorporation of α-hydroxy acids into peptides by ribosomal translation has generally been accomplished by chemical transformation of aminoacyl-tRNA after enzymatic charging [56] or by chemical tRNA acylation [15], [37], [57]. Since the α-hydroxy acids lack an α-amino group, their potential activity as AARS substrates cannot be determined by our MALDI-MS assay. We therefore studied their ability to be translated directly, by replacing one amino acid at a time with the corresponding α-hydroxy acid using the appropriate templates (*a–e*). Of the 13 commercially available α-hydroxy analogs (Ala, Asp, Glu, Gly, His, Ile, Leu, Met, Phe, Ser, Val, Trp, Tyr), only one, α-hydroxy methionine (OM), was competent in translation. Interestingly, the peptide products of translation with OM using template e (Figure 2) showed defects in initiation including lack of formylation and initiation at internal codons (Figure 7). Since α-hydroxy methionine differs in mass from methionine by only +1, we desired corroborating chemical evidence for its incorporation. When incorporated into peptides, α-hydroxy acids introduce alkali-labile ester linkages [56]. We examined the sensitivity to partial alkaline hydrolysis (Figure 7) of peptides designed to incorporate methionine between two His_6_ tags (template f). Mild hydrolysis led to cleavage of the ester-containing peptides [56], [58] and gave peptide fragments of the expected masses. As expected, treatment of the methionine containing peptide showed no cleavage.

### Fidelity of Analog Incorporation

The above experiments demonstrate that many different analogs can be incorporated into peptides at a single site. However, our ultimate goal is to prepare libraries of peptides composed primarily of unnatural AAs. The synthesis of such peptides requires that all analogs be mutually orthogonal, so that misincorporation events do not lead to a scrambling of the relationship between coding mRNA and translated peptide. Misincorporation at the level of the AARSs could occur either because an analog is charged by one or more additional synthetases, or by incorporation of contaminating natural amino acids in place of the analog. We therefore decided to begin by testing each analog in the presence of the other 19 natural amino acids and their synthetases, using an mRNA template coding for the incorporation of all 20 building blocks. This assay can detect many of the potential misincorporation events that would not be seen in the short templates used above for single-analog translation assays, since not all the competing amino acids and synthetases were present.

In order to carry out this fidelity assay, we designed template *h*, which contained codons for each of the 20 amino acids and a C-terminal His-Tag. His analogs were tested in the similar template *i*, which contains a C-terminal FLAG tag. We tested all analogs that had >25% translation efficiency as measured with templates *a–e*, using templates *h* or *i* in the presence of the other 19 natural amino acids and their synthetases. As above, we performed three experiments for each analog, a positive control with the cognate natural amino acid, a negative control with no amino acid added, and finally the experiment with the unnatural amino acid. Interestingly, in nearly all cases, the negative control experiment lacking a particular AA led to the synthesis of significant amounts of full-length peptide. The identity of these unexpected full length products varied depending on the AA withheld. For certain amino acids, peptides resulting from natural amino acid contamination (Ile, Glu, Asp) were observed. In other cases, specific misincorporation products were generated (e.g Trp→Phe, Tyr→His, and Leu→Gln/Lys, Val→Leu/Ile). Since the yield of these peptides was in most cases as high as the positive control containing 20 natural amino acids, we focused on the MALDI-MS analysis of the peptide products synthesized in the presence of an unnatural amino acid.

**Figure 8:**
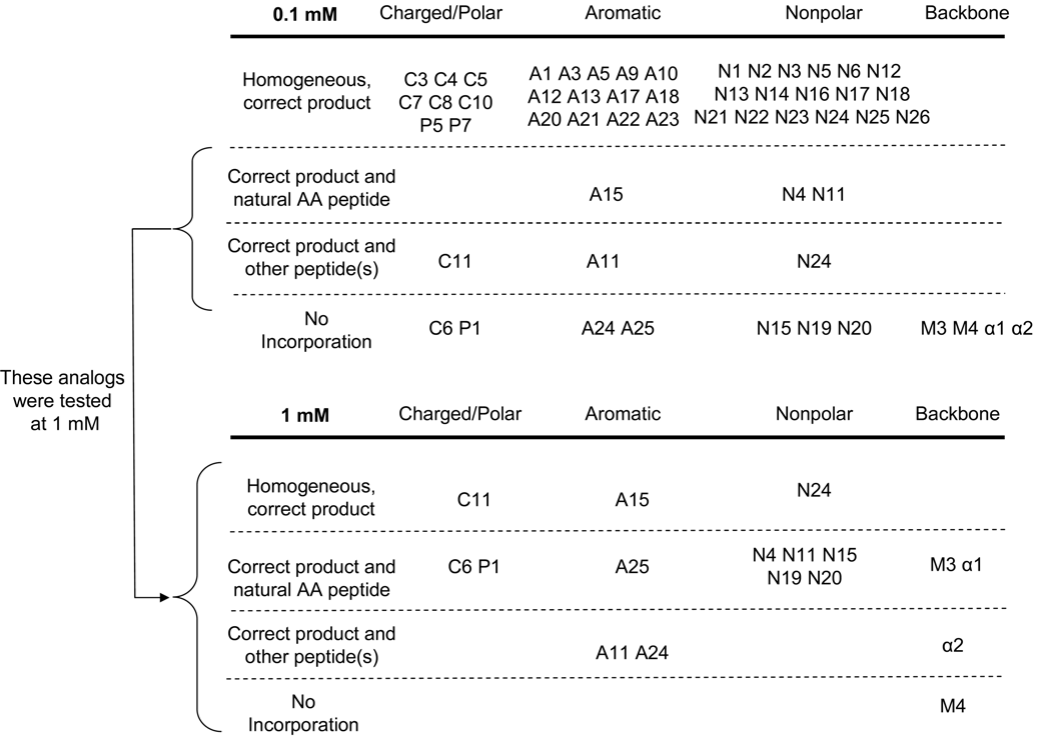
Analog incorporation efficiency and fidelity in the presence of the other 19 natural amino acids and their AARSs. Reactions used templates *h* and *i* and each reaction tested one unnatural amino acid analog. Analogs that yielded non-homogeneous peptide products at 0.1 mM, were tested again at 1 mM.

Many of the analogs were incorporated efficiently in the presence of the other natural amino acids (Figure 8). Some analogs showed two (or more) peaks in the resulting peptide MS, one of the correct mass and one or more of other masses. These analogs were categorized into two subgroups based on the identity of the other peptides observed. In some examples (A15, N4 and N11), these other masses were the natural amino acid-containing peptides, suggesting that at the concentration tested (100 µM) these analogs could only partially compete with contaminating natural AAs. In other cases (C11 and A11) the undesired peptides corresponded to peptides that resulted from other misincorporation events, some of which could not be identified by mass. A third group of amino acids showed no incorporation under these conditions.

The heterogeneous products observed with some of the analogs suggested that competition is occurring between the analog and one or more natural amino acids. We therefore re-tested all of analogs that gave heterogeneous products, as well as those that did not lead to measurable incorporation, at a 10-fold higher concentration (1 mM). At 1 mM all of the analogs except M4 showed improved incorporation into peptides including two (A1 and C11) that now gave entirely homogeneous products (Figure 8).

### Translation of a peptide containing multiple analogs

**Figure 9:**
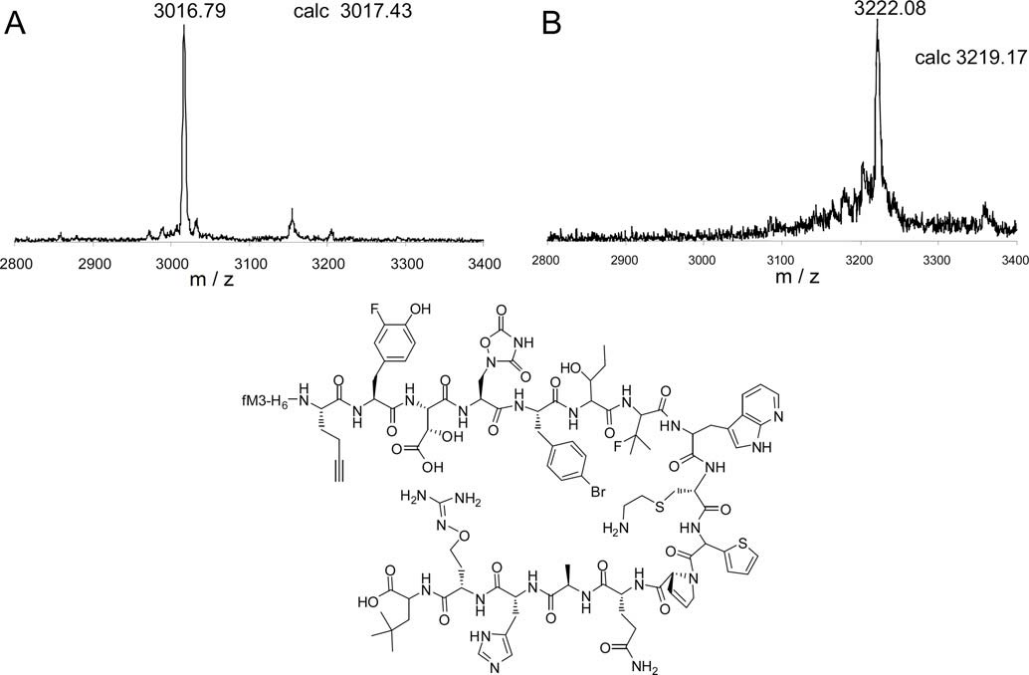
Incorporation of multiple unnatural AAs into a single peptide. (A) Mass spectrum of the peptides produced by in vitro translation of template *g* with all 15 natural amino acids. (B) Mass spectrum of the peptides produced from in vitro translation of template *g* with 13 unnatural and 2 natural amino acids. (C) Structure of the unnatural peptide that is the major product of in vitro translation of template *g* with unnatural amino acids.

The synthesis of peptides containing multiple unnatural amino acids requires a high fidelity of mRNA-directed incorporation, but in addition, the cumulative inefficiencies associated with each analog must not result in substantially diminished translation yields. We examined both of these requirements by translating peptides containing increasingly large numbers of different analogs. Prior this study, we were able to prepare peptides containing 12 different unnatural amino acids previously shown to incorporate efficiently [1]. For the experiments described here, we focused as much as possible on new analogs that were uncovered with our AARS assay [11]. We selected analogs that were efficiently incorporated in the 20 AA templates (*h* and *i*) at 1 mM as well as A24 which was chosen as an alternate to the well described Ile analog N16, and C6, which we had shown previously to be incorporated and is our only Asp analog [1]. We were able to prepare peptides of the correct mass within error using 13 different unnatural amino acids using template *g* (M->N1, Y->A3, D->C6, E->A15, F->A10, T->P5, V->N12, W->A18, K->C7, I->A24, P->N23, R->C10, L->N18) (Figure 9). Five of these, A15, N12, A24, N23, and N18 are new analogs not previously incorporated translationally into peptides. Although the products of this translation reaction are not completely homogenous, the correct peptide is the dominant product. This level of purity should be sufficient for in vitro selections of unnatural peptides using mRNA display.

Unfortunately, attempts to simultaneously incorporate 15 different unnatural AAs (analogs for every natural AA except, Asn, Ala, Ser, Gly, Cys) did not result in peptides of the correct mass. We suspect that this failure was caused by incomplete AARS/AA orthogonality and by the presence of very small amounts of the highly efficient natural amino acids in the translation mix.

## Discussion

### Correlation between aminoacylation and translation assay

**Figure 10:**
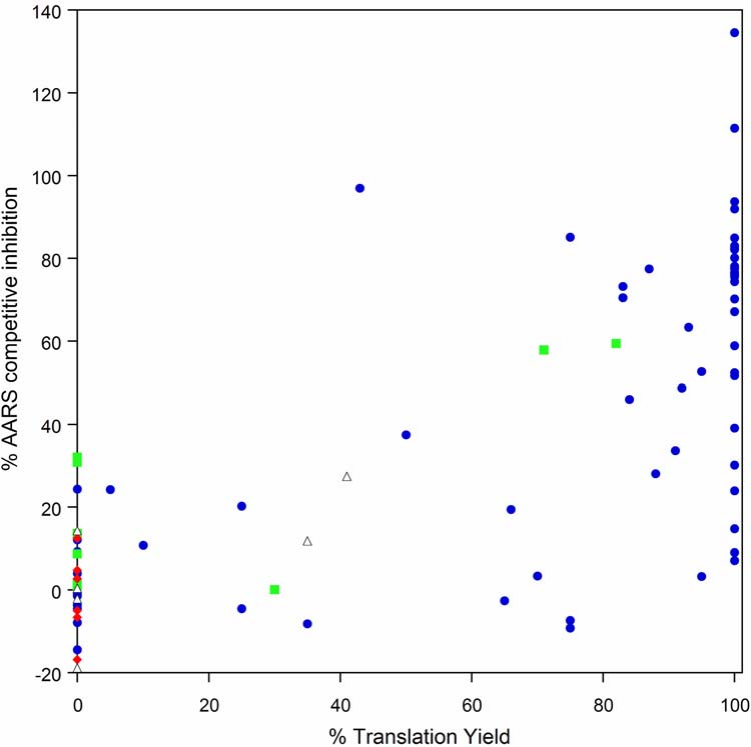
Comparison of % AARS competition and % translation yield for all of the analogs shown in Table 1. The symbols are as follows: blue circles—side chain analogs, green squares—*α,α* disubstituted amino acids, black open triangles—*N-*methyl amino acids, red diamonds–*β*-amino acids. Translation yields for analogs that gave multiple peaks in the translation experiment were set to zero for this graph.

A scatter plot comparing inhibition of the AARS reaction containing the cognate, natural amino acid and translation yield (single incorporation in templates *a*–*e*) is shown in Figure 10. Analogs that are efficient AARS competitors are located in the top half of the plot, and analogs that are translated efficiently appear on the right. From the plot it is clear that there is a correlation between competition with the natural AA for the AARS and translation efficiency—there are no highly efficient AARS competitors that translate poorly and thus would appear in the upper left quadrant of the plot. There are, on the other hand, a few analogs that translate well, but are inefficient AARS competitors and appear in lower right quadrant. These include A19, A21–A25, C9, C11, N11, N20, and P1. Several of these (A24, A25, C11, N11, N20, and P1) were among the more inefficient analogs with the 20 AA template experiments. Thus it seems reasonable to conclude that for these analogs the inefficiency is occurring during the AARS charging step. The synthetases (LeuRS and TrpRS) activating the remaining analogs in the lower right quadrant of the graph (A19, A21–23 and C9) have especially low *K_m_*s (1.5 µM [59] and 12 µM [60] respectively) for their cognate AAs. This might allow them to compensate for their relative inefficiencies of charging since the analogs were tested in these particular translation experiments at 400 µM.

### Specificity of the translation apparatus

As a step towards the synthesis of large libraries of NRP-like molecules using an in vitro translation system, we have studied the tolerance of the translation apparatus to a wide variety of amino acid analogs. The translation experiments are summarized by analog class in Table 2. Most side chain analogs are efficiently incorporated into peptides, but the backbone analogs are for the most part poorly translated. Of the 71 analogs tested, 41 side chain analogs are efficiently translated with high fidelity along with the other 19 AAs, and another 8 side chain and 2 backbone analogs should be efficiently translated if contamination of the translation system with natural, cognate AAs can be removed. The remaining analogs (mostly backbone), although AARS substrates, are poorly incorporated into peptides during translation.

**Table 2:** Summary of translation results

Analog class	Total #	Translate >25%	Homogeneous^a^	Homogeneous or natural AA^b^
Charged	13	9	7	8
Polar	7	3	2	3
Aromatic	25	18	14	15
Nonpolar	26	23	18	23
Total side chain	71	53	41	49
α,α disubstituted	7	2	0	1
Beta	11	0	0	0
*N*-methyl	6	2	0	1

a Homogeneous peptide produced with template *h* or *i* (Figure 8).

b Homogeneous peptides and analogs that gave correct peptides along with the natural AA containing peptide with template *h* or *i* (Figure 8).

Considering the side chain analogs as a class, poor charging onto tRNA appears to be the most common explanation for inefficient translation. This is supported by the correlation between competitive aminoacylation inhibition (a surrogate for aminoacylation activity) and translation efficiency (peptide yield and homogeneity) (Figure 10). For certain side chain analogs this explanation is certainly correct because they have been incorporated into peptides with good efficiency using stop codon suppression systems. For example, *p*-nitrophenylalanine (A14) has been efficiently incorporated into proteins in *E. coli* using an engineered AARS [61]. Similarly β-cyclopentyl alanine (N19) [51] and L-*C*-propargyl glycine (N9) [3] have been efficiently added using *E. coli* in vitro translation systems.

The correlation between charging efficiency and translatability suggests that enzyme engineering of the AARSs to better recognize certain analogs will be an important step to improve translation yield. Alternatively, enzymatic pre-charging of the tRNAs at high enzyme and amino acid concentrations prior to addition to the translation mixture may improve yields while minimizing product heterogeneity due to competition from efficiently acylated contaminating natural amino acids.

For the backbone analogs, the relative importance of aminoacylation efficiency vs. later steps in translation varies with the type of backbone modification. In the case of the α-hydroxy acids, we cannot detect tRNA acylation directly. However, the ribosomes from *E. coli* are known to be able to generate ester linked products for the α-hydroxy analogs of Phe, Leu, Ile, Ala, [15], [51], [57], [62] and now Met. Since only α-hydroxy methionine was incorporated into peptides in our assays of 13 α-hydroxy acids, either the synthetases or EF-Tu must discriminate against the other α-hydroxy acids. Given that many α-hydroxy acids are common metabolites (e.g. glycolate, lactate, glycerate, malate) while others may be generated at low levels in cells by hydrolysis of acyl-CoA intermediates or reduction of α-keto acids, it would not be surprising if many synthetases and perhaps EF-Tu have evolved the ability to discriminate against α-hydroxy acids. If this is the case, it will be necessary to engineer modified synthetases and perhaps EF-Tu to enable the efficient enzymatic incorporation of α-hydroxy acids into peptides. Similar synthetase engineering may be needed for the *N*-methyl amino acids, which, except for *N*-methyl His and Asp were poorly incorporated into peptides in our experiments and were poor AARS competitors as well. Previous experiments show that chemically generated *N*-methyl aminoacyl-tRNAs can be used to generate methylated peptides with reasonable efficiency [15], [63], suggesting that the main block to incorporation is not at the level of EF-Tu or the ribosome. By default, poor efficiency of tRNA charging may be the limiting factor. In contrast, the β-amino acids are likely to be discriminated against by the active site of the peptidyl transferase center, possibly due to a higher pKa of the amino group [53].

### Misincorporation/Contamination

Our discovery that full length peptides resulting from misincorporation or contamination were formed when we used the 20 AA template *h* but withheld particular amino acids was surprising because we had only observed this in a few cases with our shorter templates. For example template *d* which contains both Gln and Glu codons often gives significant full length peptide when Glu is withheld; we expect that this results from contaminating Glu in the Gln stock. In the same vein, the Glu and Asp contamination products with the 20 AA template *h* most likely resulted from the Gln and Asn stocks. Consistent with this fact, lowering the concentration of Asn improved the incorporation efficiency of Asp analog C6 in this template (not shown). The misincorporation events we observed, however, require other explanations. We reasoned that misincorporation could occur either by mischarging of a non-cognate AA by a particular AARS (e.g. the Leu to Lys/Gln misincorporation could be caused by the formation Lys/Gln-tRNA^Leu^ by LeuRS) or by readthrough of a non-cognate codon by a cognate AA-tRNA pair (e.g. Gln-tRNA^Gln^ reading the Leu codon). Preliminary investigations into the Leu→Gln/Lys misincorporation suggest that it is codon misreading that is the cause, since changing the Leu codon from CUG to UUA led to misincorporation of Phe (standard codon UUU) instead of Gln (standard codon CAG). It is possible that these misincorporation events are artifacts of our in vitro translation system, or it may be that these miscoding events represent what happens in vivo when a particular AA-tRNA is depleted in cells lacking the stringent response [64].

### Initiation

Methionine analogs provide an opportunity to investigate the substrate specificity of the initiation steps of translation. Aminoacylated initiator tRNA is inspected twice prior to initiation, once by the methionyl-tRNA formyltransferase (MTF) and once by the initiation factor IF2 [65]. Using template *e* which contains both initiator and elongator methionine codons, we found several methionine analogs that caused defects in initiation as evidenced by the production of peptides that lacked the N-terminal Met analog altogether, and appeared to either incorporate histidine in place of methionine, or to initiate at internal sites. Since our translation system lacks both the peptide deformylase and the N-terminal methionine aminopeptidase enzymes, the N-terminal residue cannot be removed posttranslationally, and therefore these peptide products must result from a failure of the initiation steps. Such translations were generally low yielding, but β-cyclopropyl alanine, N11, and α-hydroxy methionine (OM) are exceptions to this trend. Translation reactions with N11 were high yielding, but two peaks corresponding to the full-length formylated peptide and peptide lacking the N-terminal N11 residue were observed. Thus for this particular analog, the initiation machinery (most likely IF-2) appears to be more discriminating than the elongation complexes. For OM, no formylated full-length peptide was observed at all; initiation with non-formylated OM, however, proceeded with reasonable efficiency (Figure 7).

### Genetic code coverage

We have examined the translation of analogs of 17 out of the 20 canonical amino acids–all except Gly, Ser, and Cys. For 15 of these 17, we have identified at least one analog that is translated efficiently. These amino acids correspond to 47 of the 64 codons, or reassignment of 73% of the genetic code. Cumulative inefficiencies have so far limited us to the simultaneous reassignment of 13 different amino acid codons (Figure 9). Further expansion of the monomer set that is compatible with enzymatic aminoacylation and ribosomal translation may be achievable in several different ways. Clearly, the continued generation of mutant AARSs with altered substrate specificities will greatly facilitate this program. An alternative strategy is to use a combination of enzymatic, chemo-enzymatic and chemical synthesis of pre-charged tRNAs. The use of pre-charged tRNAs greatly reduces the opportunities for mis-incorporation events during peptide synthesis, but also reduces the yield of translated peptide since aminoacylated tRNA is not continuously regenerated during the course of the translation reaction. However, this factor may be less important in the synthesis of large mRNA-display or ribosome-display libraries, where each ribosome is only required to synthesize one peptide and turnover is not needed [66]–[68].

### Analog properties

**Figure 11:**
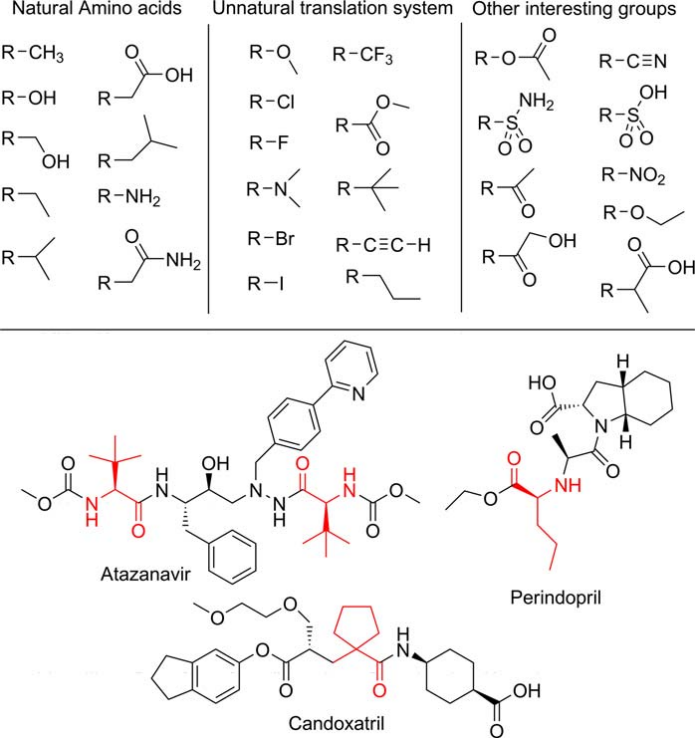
Functional groups found in known drugs and examples of drugs containing them.

It is our desire to prepare peptides using *in vitro* translation that are as drug-like as possible. To this end, we compared the side chain functional groups available to our system with a list of the most common functional groups in known drugs [69]. Of the top 46 functional groups found in drugs, 9 are represented within the natural amino acid side chains (Figure 11). Using only the analogs that are >75% efficient in our system, we can incorporate eleven more drug-like functional groups including methyl ethers, halogens, the dimethyl amino group, methyl esters and the *t*-butyl group (Figure 11). Drugs containing building blocks or functional groups present in our library include the HIV protease inhibitor Atazanavir which contains two *t*-butyl glycines (N13), Perindopril, an ACE inhibitor which includes norvaline (N4), and the antihypertensive drug Candoxatril which includes a cyclopentyl carboxylic acid moiety like that of α1. Other functional groups including acetates, nitriles, sulfonates, nitro groups, and ketones are targets for future unnatural translation building blocks.

Access to new functional groups that are found in current drugs is not enough to bestow drug-like qualities onto small molecules. These functional groups must be placed on a backbone or scaffold in precise orientations so that they can interact tightly with their targets. Coupling in vitro translation with mRNA-display [66] or ribosome-display will allow selection from extremely large libraries to find the rare peptides that properly orient these drug-like functional groups. Rigidifying the library scaffold by cyclization [58] should also improve affinity and specificity. We hope that further work aimed at increasing the building block diversity accessible through ribosomal peptide translation will enable a new path to drug discovery.

## Materials and Methods

*Amino acids* Amino acid stocks were prepared at 10 mM concentration or their maximum solubility (always >1 mM) and were stored at –20°C. For translation assays the pH of the stocks was adjusted with KOH to 7–7.5, and the stocks were filtered. Supplier information for the amino acids has been described previously [11]. The α-hydroxy acids were purchased from Fluka (OF, OM, OS, OV), Sigma/Aldrich (OA, OP, OG, OH, OI, OL, OW, OY) or City Chemical (OE).

*Protein purification* AARSs and translation factors were prepared as His-tagged constructs as previously described [1], [11].

*Quantitative AARS Assays* Each assay contained 40 mM HEPES-KOH pH 7.4, 17 mM MgCl_2_, 45 mM KCl, 3.4 mM BME, 6 mM ATP, 6% glycerol, 0.02 units/mL yeast inorganic pyrophosphatase, 50–350 µM *E. coli* total tRNA (Roche), 0.09 mg/mL BSA, and various AARS, natural amino acid and analog concentrations (Table 3). For each analog inhibition experiment three different assays were performed (A, B, and C). These assays were then mixed together (Mixing protocols 1–3) to determine differences between the ratios of the natural AA [M]^+^ and [M+15]^+ ^peaks in the presence of the analog.

**Table 3:** AARS and AA concentrations in competition assays

AARS	AARS conc (nM)	Nat AA conc (µM)	Analog conc. (mM)
AlaRS	3000	50	50
ArgRS	480	20	20
AspRS	500	50	50
GlnRS	500	50	50
GluRS	500	10	10
HisRS	500	15	15
IleRS	220	10	10
LeuRS	500	30	30
LysRS	560	10	10
MetRS	1100	50	50
PheRS	320	5	5
ProRS	500	50	50
ThrRS	950	50	50
TrpRS	500	30	30
TyrRS	580	5	5
ValRS	1000	50	50

**Assay A**: Only the natural amino acid was included in this charging assay at the indicated concentration (Table 3). The assay was initiated by the addition of the appropriate AARS and was incubated for 30 min at 37°C. A solution of 3 M NaOAc pH 5.2 (0.1 vol., 2.5 µL) was added and the assay solution extracted with unbuffered phenol:CHCl_3_:isoamyl alcohol (25:24:1), and then CHCl_3_. A portion (45 µL) of the final aqueous layer was removed and precipitated with EtOH (3 vol.) and 3 M NaOAc pH 5.2 (0.1 vol.) at –20°C. The resulting pellet was washed twice with 70% EtOH (250 µL each), allowed to dry at room temperature (∼5 min), and dissolved in 200 mM NaOAc pH 5.0 (12.5 µL). Half of the solution was frozen at –20°C, and the remaining half was added to water (3.75 µL), a freshly prepared solution of 4-formylphenoxypropyl triphenylphosphonium bromide [70] (each 69 mM in MeOH, 12.5 µL), and freshly prepared NaCNBH_3_ (200 mM in 50 mM NaOAc pH 5.0, 2.5 µL). The solution was placed in a tumbler at 37°C (occasionally additional MeOH (1–2 µL) was required for complete dissolution). After 2 hours, 4.4 M NH_4_OAc pH 5.0 (0.2 vol., 5 µL) was added to quench and the assay was mixed as described below (Mixing protocol).

**Assay B**: Assay B followed the identical procedure, except the deuterated aldehyde 4-formylphenoxypropyl triphenylphosphonium-*d_15_* bromide [11] was added in place of the non-deuterated aldehyde.

**Assay C**: Assay C included both the natural amino acid and the analog at the specified concentrations (Table 3) and used the deuterated aldehyde 4-formylphenoxypropyl triphenylphosphonium-*d_15_* bromide. The contents of the three assays are summarized in Table 4.

**Table 4:** AARS assay contents

Assay	Natural AA	Analog AA	H_15_ label	*d*_15_ label
A	+		+	
B	+			+
C	+	+		+

**Mixing protocol #1** (used to determine the ratio of the [M]^+^ and [M+15]^+^ peaks when no unnatural AA competitor was present). After the NH_4_OAc quenching of the reductive amination step, 25 µL of Assay A and 25 µL of Assay B were mixed together and the combined assays were precipitated with EtOH (3 vol.). The pellet was washed with 70% EtOH (2X) and 100% EtOH (2X) and was allowed to dry at room temperature. The dry pellet was then dissolved in 200 mM NH_4_OAc pH 5.0 (2.25 µL) and 1 U/µL Nuclease P1 in 200 mM NH_4_OAc pH 5.0 (∼0.25 µL) was added. After 20 min at room temperature, an aliquot (1 µL) was removed and added to a saturated solution of CHCA (α-cyano-4-hydroxycinnamic acid) matrix in 1:1 MeCN:1% TFA (9 µL). An aliquot (1 µL) of the resulting suspension was added to a MALDI plate and analyzed.

**Mixing protocol #2** (used to determine the ratio of [M]^+^ and [M+15]^+^ that would be found if complete, 100%, inhibition by the analog was observed). For this protocol, assay A was used alone without mixing and treated exactly as described for Mixing protocol #1.

**Mixing protocol #3** (used to determine the ratio of [M]^+^ and [M+15]^+^ when the analog was present). For this protocol, Assays A and C (25 µL each) were mixed and treated exactly as in Mixing protocol #1.

*Data analysis* After software baseline subtraction, the ratio of peak heights for derivatized natural AA-AMP and *d*_15_-AA-AMP was determined for the assay mixtures described above. To get the final % inhibition, the following equation was used % inh = (ratio A/C mix – ratio A mix)/(ratio A/B mix – ratio A mix)*100. For MetRS, ValRS, IleRS, GlnRS, GluRS, and AspRS analogs the % inhibition value was instead calculated with a calibration curve determined by mixing known ratios of d_15_-AA-AMP and H_15_-AA-AMP [11].

*Translation Assay* Translation reactions (50 µL) were carried out as previously described [1]. The mRNAs used for the translation reactions are described in the supporting information (Figure S1). For a particular template only the required natural or analog amino acids (400 µM each) and AARSs (0.1 µM MetRS, 0.3 µM LeuRS, 0.6 µM GluRS, 0.2 µM ProRS, 1.0 µM GlnRS, 1.0 µM HisRS, 0.3 µM PheRS or 0.25 µM PheRS A294G, 1.5 µM TrpRS, 0.2 µM SerRS, 0.2 µM IleRS, 0.4 µM ThrRS, 0.6 µM AsnRS, 0.6 µM AspRS, 0.5 µM TyrRS, 0.5 µM LysRS, 0.4 µM ArgRS, 0.2 µM ValRS, 0.2 µM AlaRS, 0.5 µM CysRS, 0.06 µM GlyRS) were added.

*Multiple Analog template translation* Peptide synthesis was carried out with the following optimized amino acid concentrations: N1 (800 µM), A3 (1600 µM), C6 (800 µM), A15 (1600 µM), A10 (400 µM), P5 (800 µM), N12 (3200 µM), A18 (800 µM), C7 (400 µM), A24 (800 µM), N23 (800 µM), C10 (400 µM), N18 (1600 µM), Ala (400 µM), Gln (400 µM).

*α-hydroxy methionine peptide cleavage assay* Translation reactions (100 µL) were performed as described [1] using mRNA template *f*. After elution from the Ni-NTA resin (Qiagen) into 0.2% TFA (50 µL), the peptides were treated with 1 M NaOH (15 µL) for 60 min at 37°C. The assays were quenched with 10% TFA (6 µL) and 1 M HOAc (5 µL) and were purified by Zip-Tip C_18_ chromatography as described above. The products were analyzed by MALDI-TOF MS.

## Supporting Information

Figure S1 (0.04 MB DOC)
