# Supplementary material for: An Expanded Set of Amino Acid Analogs for the Ribosomal Translation of Unnatural Peptides
Source: PLoS One. 2007 Oct 3;2(10):e972. doi: 10.1371/journal.pone.0000972 (PMC1989143; doi:10.1371/journal.pone.0000972)
Supplement: Figure S1 — (0.04 MB DOC) [file pone.0000972.s001.doc]

----------------------------------5' UTR--------------------------------- -------------------------------3' UTR-----------------------------

5'-GGGAGACCACAACGGUUUCCCUCUAGAAAUAAUUU AGCCCGAAAGGAAGCTGAGTTGGCTGCTGCCA-3'

-------------------------S/D rbs----------------**NdeI BamHI**-------------

GUUUAACUUUAAGAAGGAGAUAUACAU**…**UCCGGCUGCUAACAA

**NdeI BamHI**

(a) AUG CAU UUU AGC UGG GAU UAU AAA GAC GAC GAU GAC AAA UAG UAG GGA

M H F S W D Y K D D D D K stop stop

FLAG affinity tag

(b) AUG GUG GCG UGC GGC GAU UAU AAA GAC GAC GAU GAC AAA UAG UAG GGA

M V A C G D Y K D D D D K stop stop

FLAG affinity tag

(c) AUG ACC AUU AAC CGU GAU UAU AAA GAC GAC GAU GAC AAA UAG UAG GGA

M T I N R D Y K D D D D K stop stop

FLAG affinity tag

(d) AUG CUG GAA CCG CAG GAU UAU AAA GAC GAC GAU GAC AAA UAG UAG GGA

M L E P Q D Y K D D D D K stop stop

FLAG affinity tag

(e) AUG GAU UAU AAA AUG CAU CAC CAU CAC CAU CAC UAG UAG GAU UAU AAA GAC GAC

M D Y K M H H H H H H stop stop

His affinity tag

GAU GAC AAA UAG UAG GGA

(f) AUG CAU CAU CAC CAU CAC CAU CUG GCG AUG GCG UUC CAC CAC CAU CAC CAU CAU

M H H H H H H L A M A F H H H H H H

UAG UAG GAU UAU AAA GAC GAC GAU GAC AAA UAG UAG GGA

stop stop

(g) AUG CAU CAC CAU CAC CAC CAU AUG UAU GAU GAG UUC ACU GUG UGG AAG AUC CCG

M H H H H H H M Y D E F T V W K I P

CAG GCG CAC CGG CUA UAG UAG GAU UAU AAA GAC GAC GAU GAC AAA UAG UAG GGA G

Q A H R L stop stop

(h) aUg caU cUg gaa ccg cag gau uau aaa uuu auu gug ucu acc gcg aac ugc ugg

M H L E P Q D Y K F I V S T A N C W

cgu ggc cac cau cac cac cau caC UAG UAG

R G H H H H H H stop stop

(i) aug cau cug gaa ccg cag gau uau aaa uuu auu gug ucu acc gcg aac ugc ugg

M h L E P Q D Y K F I V S T A N C W

cgu ggc gau uau aaa gac gac gau gac aaa uag uag

R G D Y K D D D D K stop stop

Multi-Trp template

aug caU UUa gaa ccg cag gaU UaU aaa UUU aUU gUg agc acc gcg aac Ugc Ugg Ugg

m H L E P Q D Y K F I V S T A N C W w

Ugg Ugg cgU ggc cac caU cac cac caU cac

W W R G H H H H H H

Figure S1. List of mRNAs used in the translation experiments. The sequences of the 5' and 3' untranslated regions (UTR) of mRNAs transcribed from the pET12b T7 promoter are shown with the ribosome binding site (rbs) underlined. The originating mRNA (d) was constructed by ligating complementary oligonucleotides that correspond to the entire open reading frame with the appropriate overhangs into pET12b opened with NdeI and BamHI. The codons for the FLAG tag were chosen to provide a unique PsiI site for the in-frame ligation of complementary oligonucleotides for mRNAs a-c with the with the C-terminal FLAG tag. Longer complementary oligonucleotides with stop codons prior to the C-terminal

FLAG tag were inserted into the NdeI and PsiI sites for mRNAs e-g. Complimentary oligonucleotides with stop codons were inserted into the the PsiI and BamHI sites for mRNAs h, i, and the multi-Trp template
